# Nurturing the scientific mind: resilience and job satisfaction among Saudi faculty

**DOI:** 10.3389/fpsyg.2024.1341888

**Published:** 2024-02-05

**Authors:** Ahmed M. Asfahani

**Affiliations:** Department of Human Resources Management, University of Business and Technology, Jeddah, Saudi Arabia

**Keywords:** psychological resilience, job satisfaction, motivation, well-being, quality education

## Abstract

This study examines the interplay between psychological resilience, job satisfaction and research motivation among teachers at Saudi Arabian universities. Particular attention is paid to the relationship between satisfaction and academic performance and well-being. The data is based on a survey of 321 faculty members, and descriptive statistics and correlations are used. The research instruments included a self-developed scale to measure these constructs, and data analysis was conducted using SPSS software. The findings revealed moderate job satisfaction levels, with a significant correlation between resilience and both job satisfaction and research motivation. Resilience was identified as a key predictor of job satisfaction, especially among professors compared to lecturers. Uniquely focused on the Saudi academic context, this study offers insights into culturally specific factors affecting academic faculty, underscoring the importance of enhancing resilience and satisfaction within academic settings. These implications align with Saudi Arabia’s Vision 2030 goals, suggesting targeted strategies to improve faculty well-being and performance.

## 1 Introduction

In the multifaceted and dynamic milieu of higher education, the confluence of emotional, cognitive, and behavioral dimensions is pivotal in shaping the experiences of faculty members. This is particularly evident in relation to fundamental constructs such as job satisfaction, psychological resilience, and motivation towards scientific research (Mgaiwa, 2021; Brogden et al., 2022; Hanson et al., 2022). In an era marked by rapid globalization and technological advancement, the need to understand their interplay assumes heightened significance, especially as traditional educational paradigms in the Middle East converge with contemporary global standards (Alsharari, 2020). Furthermore, higher education institutions assume a critical role that transcends their contributions to industry and commerce; they are instrumental in fostering an innovative workforce and in the cultivation of individuals who markedly influence societal progression (Sagr, 2023). Consequently, it is imperative for these institutions to actively cultivate environments conducive to academic growth and the optimal utilization of talent (Howaniec et al., 2022). This not only attracts but also retains distinguished academics. In this context, the resilience and job satisfaction of faculty members are pivotal, influencing not only their personal well-being but also the effectiveness and quality of higher education (Mansfield, 2020; Nicol and Bice, 2022).

Saudi Arabia, a nation now undergoing significant social and economic changes, relies heavily on its higher education system as a catalyst for advancement and the generation of innovative ideas (Sagr, 2023). The ability of the academic community to adapt, innovate, and contribute to social changes is strongly influenced by work satisfaction and resilience (Alfawaz et al., 2021). Moreover, the impetus behind scientific inquiry, which is crucial for the progress of a nation, influences its ability to compete in the global knowledge-based economy (Alsharari, 2020). Hence, the significance of upholding academic progress in Saudi Arabia cannot be overstated, as it plays a crucial role in achieving the needed advancements across diverse sectors, aligning with the aspirations outlined in Vision 2030 (Singh et al., 2022). Hence, it is imperative to establish conducive work environments within Saudi higher education institutions in order to effectively retain faculty members, enhance their job motivation, and assist them in navigating various challenges (Kuwaiti et al., 2020). This includes fostering the development of psychological resilience, which can potentially augment their overall job satisfaction (Nagoji and Mackasare, 2023).

Despite extensive research on faculty job satisfaction (Szromek and Wolniak, 2020), psychological resilience (Kowler et al., 2023), and motivation within academic contexts (Daumiller et al., 2020), significant gaps persist, particularly in the context of Saudi Arabian universities. Most existing studies, such as those by Sessions et al. (2023) and Boamah et al. (2023), either focus on individual aspects or overlook the dynamic interplay between these factors, especially within Saudi Arabia’s unique socio-cultural setting. This oversight is critical, considering the rapid socio-economic changes and evolving educational policies in the region. Furthermore, the applicability of Western-developed models in the Saudi educational context remains questionable. Studies by Hanson et al. (2022), Stupnisky et al. (2023), Turgumbayeva et al. (2019), and Marcus et al. (2022) highlight the underexplored regions, particularly how resilience and job satisfaction collectively influence research motivation and the relevance of motivational theories like self-determination theory (SDT) and social cognitive career theory (SCCT). Addressing these gaps, our study seeks to provide a more holistic understanding of these interactions, aiming to enrich academic discourse and inform policy-making and institutional practices in Saudi Arabian universities. Ultimately, this research is pivotal in adapting educational systems to better align with the evolving needs of their faculty in a culturally nuanced context.

Utilizing a descriptive and correlative research design, this study delves into the relationships between psychological resilience, job satisfaction, and research motivation among faculty in Saudi Arabian higher education. It examines the relationships between resilience, job satisfaction, and research motivation and assesses the influence of faculty rank and departmental structures. Aimed at aligning with Saudi Arabia’s Vision 2030, the study offers insights for policymaking and faculty development, providing a nuanced understanding of how cultural and institutional factors shape academic experiences. This approach is instrumental in formulating strategies for educational enhancement in the region.

This research starts with a literature review and advances through a detailed methodology of research design and data collection, analyzing survey data on faculty members in Saudi Arabian universities. It places these findings within the broader academic and socio-cultural context of Saudi Arabia, addressing study limitations and proposing future research directions. The conclusion underlines key insights and their implications for educational policy and faculty experiences in higher education, ensuring a cohesive build-up and enriching the study’s contributions to both academic research and practical application.

## 2 Literature review

### 2.1 Saudi educational dynamics

In the ever-evolving academic landscape of Saudi Arabia, the dynamic interplay of cultural, social, and economic factors is reshaping academic experiences. These influences are particularly evident in job satisfaction, resilience, and motivation within academia. The study by Zidi and Al-Shallaqi (2023) has played a pivotal role in underscoring the increasing importance of social relations and gender inclusivity. Their findings suggest a cultural pivot towards enhanced interpersonal dynamics and more balanced gender representation in academia. Meanwhile, Sagr’s (2023) study delves into the cultural impacts on academic resilience, pointing out the growing emphasis on innovative educational environments and exploring faculty turnover intentions, revealing the complexities of melding traditional cultural values with contemporary organizational demands.

A notable tension between traditional methods and modern educational advancements emerges in the works of Alqahtani and AlNajdi (2023). Their research on the hesitancy towards new technologies like augmented reality contrasts with Aldarmahi et al.’s (2022) findings on the efficacy of preparatory programs. This juxtaposition illustrates the challenges faced in integrating progressive educational tools within established pedagogical frameworks. Additionally, Alomaish et al. (2022) shed light on medical students’ specialization choices, influenced by societal values favoring patient-interactive fields, thereby underscoring the impact of cultural preferences on career decisions. The shift towards a more student-centered learning approach is further highlighted by Sabbagh et al. (2020). They emphasize the evolving perceptions of the educational environment and the need for adaptability to meet students’ requirements. Alkathiri (2020) offers a critical assessment of Saudi higher education, pointing out a lag in adopting 21st-century educational practices and a mismatch between current methodologies and student expectations.

In the Saudi higher education system, faculty experiences are shaped by a complex interplay of traditional educational values and modern global standards. Kuwaiti et al. (2020) emphasize the impact of administrative policies and conventional teaching methods on faculty job satisfaction. Alnasser and Almoaily (2022) bring attention to language policy inconsistencies, reflecting broader systemic challenges. Safadi and Vlachopoulos (2021) point out gaps in faculty development and perceptions of quality assurance, signaling a disconnect between policy implementation and practical application.

The integration of technology into education, especially during the COVID-19 pandemic, as noted by Alnasib’s (2023) study on AI readiness, presents a mixed bag of challenges and opportunities for faculty. Alharbi and Albelihi (2023) highlight struggles in research, publication, and curriculum integration, further complicating the faculty experience. Collectively, these studies paint a picture of a Saudi higher education system at a critical juncture, calling for strategic policy reforms and enhanced faculty development. Such changes are necessary to balance rich traditions with the demands of modern educational trends and technologies, thereby improving faculty job satisfaction, resilience, and motivation in a rapidly changing educational landscape.

### 2.2 Theoretical framework

Understanding faculty job satisfaction and resilience within the context of Saudi Arabian universities necessitates an integrative approach. This theoretical framework combines pivotal theories, each offering distinct yet complementary insights into the multifaceted nature of faculty experiences amidst the unique cultural, economic, and policy landscapes of Saudi Arabia’s rapidly evolving academic environment.

Herzberg’s two-factor theory forms the initial lens, distinguishing between intrinsic motivators and extrinsic hygiene factors (Herzberg, 1959). Its capacity to dissect the complex interplay of factors influencing job satisfaction is especially relevant in Saudi Arabia, where aspects such as cultural norms, recognition, and working conditions differentially impact faculty satisfaction (Adinew, 2023). However, a limitation of this theory in a diverse cultural context like Saudi Arabia is its Western-centric perspective, which might overlook nuanced cultural factors in job satisfaction (Alrawahi et al., 2020). Maslow’s hierarchy of needs broadens the perspective, suggesting that satisfaction and motivation stem from fulfilling both basic and higher-order needs (Maslow, 1943). In Saudi Arabian universities, this framework is crucial for assessing how these institutions cater to diverse needs, considering cultural differences in the perception of these needs (Hailat et al., 2021).

The Job Characteristics Model (JCM) posits that job design plays a crucial role in enhancing satisfaction (Hackman and Oldham, 1975), particularly relevant under the ongoing educational reforms in Saudi Arabia (Singh et al., 2022). This perspective aligns with Self-Determination Theory (SDT), which identifies autonomy, competence, and relatedness as fundamental to faculty well-being (Deci and Ryan, 2012; Haw et al., 2023). In this context, the way these psychological needs are addressed or neglected, considering institutional and cultural factors, becomes pivotal in influencing faculty motivation and satisfaction (Weinstein et al., 2023).

Moreover, the social-cognitive career theory (SCCT) offers insights into how personal beliefs, environmental factors, and behaviors interact to shape faculty career paths and satisfaction (Lent et al., 1994; Toropova et al., 2021). Particularly in Saudi Arabia, this theory helps understand the dynamic relationship between individual goals and the evolving educational landscape (Alenezi, 2023). Lastly, Affective Events Theory (AET) underscores the significance of emotional responses to workplace events in determining job satisfaction and resilience (Weiss and Cropanzano, 1996; Beuren et al., 2022). This theory is especially pertinent in Saudi universities, where understanding the emotional dynamics and cultural nuances of emotional expression and workplace interactions is key to grasping their impact on faculty job satisfaction and resilience (Murdock, 2022). This comprehensive approach, blending these theories, offers a multi-dimensional perspective on faculty satisfaction in the Saudi academic environment.

### 2.3 Job satisfaction in academia

Job satisfaction within academia, inherently multifaceted and dynamic, interweaves emotional, cognitive, and behavioral dimensions. This construct, as outlined by Jameel and Ahmad (2020), stems from a positive emotional state linked to diverse job facets, with personal fulfillment and autonomy as pivotal elements. Highlighting transitions, Laari et al. (2021) reveal these drivers’ prominence during professional shifts. Crucial in academia’s landscape is the balance between teaching and research, as Ghasemy et al. (2022a) stress, alongside factors like work hours and professional development opportunities, emphasized by Hong (2020), shaping job satisfaction. Cultural and religious contexts, explored by Mousa and Chaouali (2022), add complexity, showing job satisfaction transcends financial incentives or career advancement, intertwining with intellectual fulfillment. The AET, applied by Ghasemy et al. (2022b) in academia, underscores the unique blend of dynamics at play.

Integrating Herzberg’s two-factor theory, Maslow’s hierarchy of needs, and the JCM offers diverse perspectives on faculty satisfaction. Herzberg’s theory, supported by Basbeth et al. (2021), balances intrinsic motivators against extrinsic hygiene factors, influencing satisfaction. However, Ali et al. (2021) point out intrinsic satisfaction’s complexity, especially during the COVID-19 pandemic. Complementing this, Maslow’s model, applied in Lee et al.’s (2021) research, proposes a hierarchical approach to faculty satisfaction, focusing on psychological needs yet potentially oversimplifying academic roles. The JCM, used in Hanson et al. (2022), emphasizes job autonomy and professional development as key drivers but may have limitations in diverse contexts. Stupnisky et al. (2023) suggest integrating intrinsic motivation, psychological need fulfillment, and intellectual stimulation for satisfaction. Yet, Berkowitz et al. (2021) highlight a gap in these models in addressing evolving academic roles, underscoring the need for dynamic, integrative models that reflect modern academia’s complexities.

In higher education, job satisfaction arises from the interplay of intrinsic, extrinsic, and environmental factors. Studies like Tran and Do (2020) and Strong et al. (2021) emphasize intrinsic motivators such as academic freedom and personal achievement, counterbalanced by the stress and burnout noted by Singh et al. (2022). Extrinsic factors, including salary and job security, crucial in Rothacker-Peyton et al. (2022), highlight remuneration and workplace safety as satisfaction determinants. The COVID-19 pandemic, as DeDiego et al. (2023) discuss, has amplified these challenges, affecting job security and the shift to online teaching. Environmental aspects, such as organizational culture and leadership, significantly shape job satisfaction, with Cone and Unni (2020) emphasizing the importance of faculty involvement in decision-making. This intricate relationship underscores a balanced approach to addressing the diverse factors contributing to academic job satisfaction. Therefore, the researcher hypothesizes the following based on the preceding discussion:

*Hypothesis 1*: Saudi Arabian higher education faculty members are likely to experience a moderate level of job satisfaction.

In the realm of academic job satisfaction, a nuanced understanding emerges when considering the varying priorities and perceptions across different ranks, such as lecturers and professors. A study by Qawasmeh et al. (2024) highlights the crucial role of human resources management in shaping job satisfaction, suggesting that while lecturers might value developmental opportunities, professors often prioritize recognition and job security. This distinction is further elaborated by Mgaiwa (2021) and Spasovski and Pecakovska (2020), who focus on the impact of the work environment, revealing that supportive conditions significantly enhance job satisfaction, with lecturers perhaps more sensitive to these factors due to their typically earlier career stages. Additionally, Shrestha (2019) introduces the age factor, implying that job satisfaction increases with age and experience, a trend likely more apparent in professors than lecturers. Furthermore, Mohammadi and Karupiah (2020) shed light on the differing emphases on financial rewards and job security between academic ranks, indicating a higher priority for financial stability among lecturers as opposed to professors. Consequently, this comprehensive analysis underscores the need for academic institutions to adopt differentiated strategies for addressing job satisfaction, acknowledging the diverse needs and expectations inherent in each academic rank. Therefore, the researcher hypothesizes the following based on the preceding discussion:

*Hypothesis 2*: There is a significant difference in job satisfaction between lecturers and professors, stemming from their distinct roles and responsibilities in academia.

### 2.4 Psychological resilience in academic settings

Psychological resilience in academic contexts is a multifaceted construct that is crucial for navigating the inherent challenges and pressures of academic environments. Nicol and Bice (2022) define resilience as the capacity of individuals and institutions to adapt and thrive amidst adversities, such as pandemics, emphasizing its necessity for maintaining educational quality and personal well-being in crisis situations. Expanding upon this, Brogden et al. (2022) consider community and institutional resilience, underscoring the importance of a transdisciplinary approach in sustainability education and the significance of research and innovation capacities in higher education institutions for disaster resilience. This broader perspective is complemented by Mansfield (2020), who focuses on teacher resilience, exploring its forms and implications in educational settings.

Building on these diverse viewpoints, the literature underscores resilience as a dynamic, context-specific process of adaptation and positive response to adversity, essential in both crisis and routine academic scenarios. However, a dichotomy emerges between individual and systemic resilience, suggesting a need for integrative models that combine both aspects, particularly in academic settings where they are equally crucial. Moreover, recent studies by Mead et al. (2021), Gull et al. (2023), and Durso et al. (2021) emphasize adaptability and flexibility as key resilience components, especially significant in adapting to rapid changes like digital transformation in higher education. This emphasis on adaptability seamlessly connects to the importance of robust support systems, as highlighted by Shaik et al. (2022) and Moore et al. (2023), who point out the role of communities of practice, mentorship, and faculty support in bolstering personal resilience during crises. Additionally, the aspect of emotional and mental resilience, discussed by Kowler et al. (2023), emerges as crucial for maintaining mental health and emotional well-being under academic pressures.

Transitioning to the organizational perspective, Tobin and Taff (2020) show that institutional policies and culture significantly impact resilience, thus creating environments conducive to its development. In tandem with organizational factors, individual traits like self-control and personal organization, as noted by Okwuduba et al. (2022), are integral to the resilience framework. However, these studies collectively reveal a research gap in addressing the diverse needs of various academic populations, including minority and non-traditional students and faculty, calling for more nuanced research and policy development focused on nurturing resilience across different demographic groups in academic settings.

Furthermore, the relationship between resilience and job satisfaction in academia is multifaceted and complex. Sessions et al. (2023) find that resilience enhances teaching effectiveness, especially under challenging circumstances like the COVID-19 pandemic. Conversely, Boamah et al. (2023) highlight the adverse impact of increased workload and limited adaptability on job satisfaction, indicating a balance between resilience and occupational demands. This interplay between personal well-being and professional demands suggests a nuanced relationship between resilience, adaptability, and job satisfaction, with external support from institutions playing a crucial role in fostering resilience. While this relationship benefits adaptability and teaching effectiveness, it also presents areas for further exploration, such as the direct impact of resilience on research productivity and the equilibrium between internal and external facilitators of resilience. Therefore, the researcher hypothesizes the following based on the preceding discussion:

*Hypothesis 3*: There is a significant correlation between psychological resilience and job satisfaction among Saudi faculty members.

*Hypothesis 4*: The degree of job satisfaction among faculty members in Saudi Arabia can be effectively predicted by examining their psychological resilience.

### 2.5 Scientific research motivation

In exploring research motivation within academic settings, SDT and SCCT stand out as foundational yet contrasting frameworks. SDT, with its focus on intrinsic motivations and fulfilling basic psychological needs like autonomy, competence, and relatedness, is well-represented in studies such as Hanson et al. (2022) and Stupnisky et al. (2023). These studies particularly highlight SDT’s relevance in understanding faculty motivations in teaching and research. However, the theory’s inclination towards intrinsic factors may overlook extrinsic motivations and cultural variances, a gap identified in studies by Turgumbayeva et al. (2019) and Marcus et al. (2022).

Transitioning to a complementary perspective, SCCT, as demonstrated in Sawitri and Creed’s (2021) work, weaves individual beliefs with contextual elements, shedding light on how personal motivations interact with the research environment. This model, while offering a broader lens that encompasses both intrinsic and extrinsic motivators, might not delve as deeply into internal motivational mechanisms as SDT does (Liu et al., 2016). The interplay of these theories suggests that a singular framework may be insufficient to fully grasp the diverse motivators in academic research. An integrative approach, combining elements of SDT and SCCT, could offer a more comprehensive understanding, particularly in terms of balancing intrinsic motivation with environmental and cultural factors (Jiang et al., 2023).

In academia, the nexus of motivation, productivity, and satisfaction is complex and layered. Studies by Atieno et al. (2022) and Mudrák et al. (2020) consistently highlight the importance of both intrinsic and extrinsic motivational factors in boosting academic productivity. Here, personal development, societal contributions, and rewards feature prominently. Luczaj and Kurek-Ochmanska (2021) expand this view by emphasizing the role of cultural diversity and external expertise. A pivotal study by Akyildiz and Durna (2021) shifts the focus to the psychological impacts of the COVID-19 pandemic on academic productivity, underscoring the critical role of psychological well-being in maintaining motivation. This is complemented by Spasovski and Pecakovska (2020), who illustrate how challenging work conditions and a threatened academic identity can significantly impact motivation and productivity, thus highlighting the necessity of a supportive academic milieu. Therefore, the researcher hypothesizes the following based on the preceding discussion:

*Hypothesis 5*: A significant association exists between psychological resilience and scientific research motivation within the Saudi faculty members.

Furthermore, regarding academic satisfaction, Noone and Young (2019) point to faculty morale, influenced by peer relationships and leadership perceptions. The success of writing retreats in bolstering scholarly activity and satisfaction, primarily through peer support and networking, underscores the value of collaborative environments in academia. Daumiller and Dresel (2020) add complexity to this narrative by examining the variable nature of faculty motivation across different academic activities, emphasizing the domain-specificity of motivational factors. Therefore, the researcher hypothesizes the following based on the preceding discussion:

*Hypothesis 6*: A significant relationship exists between job satisfaction and the motivation to conduct scientific research.

## 3 Methodology

### 3.1 Procedure and participants

In this descriptive and correlative study, the researcher investigated the nexus between psychological reliance, job satisfaction, and motivation for scientific research among faculty members within Saudi Arabian higher education institutions. Ethical clearance was duly obtained from the University of Business and Technology Research Ethical Committee under protocol number 30959, and informed consent was secured from all respondents, ensuring compliance with established ethical standards. Utilizing a comprehensive 114-item questionnaire disseminated through the Survey Monkey platform, a sample of 1000 faculty members was systematically selected via a simple random sampling method diverse university, ensuring a representative cross-section across various faculties and departments. Prior to the principal survey administration, preliminary research involving 100 faculty members was undertaken to ascertain and enhance the psychometric robustness of the employed scales. Contact details were acquired from institutional web portals, and invitations to partake in the 20-min online survey were disseminated, underscoring the anonymity and voluntary nature of participation. The response rate stood at 32.1%, with 321 completed surveys, surpassing the initial sample size estimation of 160 as determined via G*Power software analysis, thereby ensuring a statistical power threshold of 0.80. Table 1 presents the sociodemographic characteristics of the participants.

**Table 1:** Sociodemographic characteristics of the participants.

Sample characteristics	*N*	%
*Gender*		
Male	192	59.8
Female	129	40.2
*Age (years)*		
25–34	62	19.3
35–44	151	47
45–54	80	24.9
55–64	25	7.8
65+	3	0.9
*Academic ranking*		
Professor	216	67.3
Lecturer	105	32.7

*N* = 321, source: the author.

### 3.2 Instruments

In the present study, an imperative was identified to develop precise, empirically validated measurement instruments for assessing psychological resilience, job satisfaction, and research motivation within academic settings. This necessitated the creation and subsequent validation of three distinct scales, each meticulously designed to evaluate specific constructs pertinent to faculty members.

#### 3.2.1 The psychological resilience scale

An instrument encompassing 47 items was structured into three discrete dimensions: commitment (16 items), control (15 items), and challenge (16 items). The response modality for the scale ranges from “never” (denoted by one point) to “always” (three points). Analytical scrutiny of the scale, as exhibited in Table 2, involved examining correlation coefficients between individual item scores and their respective dimensional scores. These coefficients demonstrated statistical significance at the 0.01 level, thereby affirming the internal consistency of the scale. Cronbach’s alpha coefficients were calculated, revealing values of 0.762 for commitment, 0.751 for challenge, and 0.793 for control. The aggregate scale reliability was determined to be 0.886, indicative of a high degree of internal consistency.

**Table 2:** Correlation coefficients for the psychological resilience scale.

Commitment		Control		Challenge	
Statement	*r*	Statement	*r*	Statement	*r*
1	0.572	1	0.666	1	0.616
2	0.582	2	0.673	2	0.520
3	0.594	3	0.711	3	0.609
4	0.637	4	0.672	4	0.762
5	0.420	5	0.713	5	0.642
6	0.644	6	0.620	6	0.650
7	0.602	7	0.616	7	0.636
8	0.590	8	0.738	8	0.476
9	0.628	9	0.755	9	0.477
10	0.411	10	0.660	10	0.456
11	0.455	11	0.611	11	0.458
12	0.520	12	0.683	12	0.689
13	0.565	13	0.669	13	0.666
14	0.661	14	0.737	14	0.655
15	0.586	15	0.757	15	0.605
16	0.436			16	0.583

The descriptive analysis of 47 variables, each measured on a consistent 3-point scale, reveals a significant range in aggregated responses, with sums stretching from 402 to 843, suggesting variable levels of engagement across items. Moving to measures of central tendency, the mean scores exhibit a wide range, from 1.252 to 2.654, indicative of diverse average respondent ratings. Furthermore, the spread of responses, as gauged by standard deviations, spans from 0.598 to 0.898, underscoring a disparity in the distribution of responses that hints at varying levels of consensus among participants. This variability is mirrored in the variance statistics, which further accentuate the differential distributions around the mean values. Lastly, the standard errors vary, pointing to a range of precision in these mean scores. Collectively, these statistics paint a picture of consistent scale usage but with distinct patterns of response distribution, highlighting the multifaceted nature of the data and the complex insights they offer into the constructs being measured.

#### 3.2.2 Job satisfaction scale

The study developed a comprehensive scale for assessing job satisfaction, encompassing 43 items distributed across seven salient dimensions. These dimensions include, but are not limited to, engagement in decision-making processes and distribution of teaching responsibilities. The scale employs a scoring algorithm that spans from 43 to 129, facilitating a granular analysis of job satisfaction levels. As delineated in Table 3, the reliability coefficients for each dimension were calculated, culminating in an overall scale reliability coefficient of 0.901. This evidences the scale’s capability to accurately and reliably assess the multifaceted nature of job satisfaction within academic environments.

**Table 3:** Correlation coefficients for the job satisfaction scale.

Dimensions	Number of statements	Reliability coefficient
Decision-making participation	8	0.833
Distribution of teaching responsibilities	9	0.714
Academic advising	5	0.753
Academic promotion	3	0.725
Academic interpersonal interactions	4	0.704
Work environment	8	0.711
Compensation	6	0.700
Total	43	0.901

#### 3.2.3 Scientific research motivation scale

It was conceptualized to evaluate facets of research motivation, comprising 24 statements across three dimensions: “Accomplishment Motivation,” “Ambition Level,” and “Challenge Seeking.” The response spectrum of this scale ranges from 1 (“always applies”) to 3 (“does not apply”). The scale’s construct validity was ascertained using a transactional analysis methodology with a sample of 100 participants. This analysis yielded statistically significant correlations across all components, thereby validating the scale’s construct. Reliability, as determined through Cronbach’s alpha, was found to be 0.911, illustrating the scale’s consistency and dependability in measuring research motivation.

### 3.3 Data analysis

In the data analysis phase, a comprehensive approach was employed to investigate the relationship between job satisfaction, psychological resilience, and motivation for scientific research among faculty members in Saudi Arabia. The process began with the use of a one-sample t-test to compare observed mean satisfaction levels across different job dimensions against hypothesized means, addressing the first hypothesis. This step was followed by correlation analyses to explore the relationships between psychological resilience, job satisfaction, and research motivation for the third and fifth hypotheses, examining aspects such as decision-making participation and teaching responsibilities. To further understand the dynamics between job satisfaction and scientific research motivation, *t*-value analyses were conducted, providing insights into these relationships. Additionally, two-sample independent t-tests were utilized to investigate differences in job satisfaction levels between university professors and lecturers, focusing on the second hypothesis. Finally, the analysis culminated with a simple linear regression ANOVA test and stepwise multiple regression analysis to determine the predictive power of psychological resilience on job satisfaction, thereby addressing the fourth hypothesis.

## 4 Results

*Hypothesis 1* was rigorously examined using a one-sample t-test, as delineated in Table 4, to determine whether faculty members in Saudi Arabia exhibit moderate levels of job satisfaction across various dimensions. The empirical findings reveal that the observed mean satisfaction levels in key areas, decision-making participation (15.873), teaching responsibilities (17.931), academic advising (10.240), academic promotion (6.140), and work environment (16.187), were in close proximity to the respective hypothesized means. The corresponding *t*-values, ranging from 0.127 to 1.741, did not indicate any statistically significant deviations from these hypothesized benchmarks. Furthermore, the total satisfaction level, with an observed mean of 86.280, closely aligned with the hypothesized mean of 86. These results collectively support *Hypothesis 1*, suggesting that the job satisfaction levels among the faculty members in Saudi Arabia are congruent with the proposed moderate levels, as per the hypothesis.

**Table 4:** The *T*-value associated with the job satisfaction measure.

Dimensions	Hypothesized mean	Observed mean	SD	*T*-value
Decision-making participation	16	15.873	1.206	0.127
Teaching responsibilities	18	17.931	1.868	0.568
Academic advising	10	10.240	1.740	1.689
Academic promotion	6	6.140	0.505	1.393
Interpersonal interactions	8	8.140	1.147	1.495
Work environment	16	16.187	1.178	1.741
Compensation	12	11.787	1.673	1.561
Total	86	86.280	3.738	0.917

Using two-sample independent *t*-tests, the findings presented in Table 5 provide a robust validation of *Hypothesis 2*, which posited significant disparities in job satisfaction levels between university professors and lecturers. The analysis reveals that professors consistently report higher levels of satisfaction across various job facets. In the dimension of decision-making participation, a significant difference is noted, with professors exhibiting a mean satisfaction level of 16.408 compared to lecturers’ 15.614, yielding a *t*-value of 3.967. This trend of higher satisfaction among professors extends to teaching responsibilities (mean: 18.980 vs. 17.400, *t*-value: 5.293) and academic advising (mean: 11.601 vs. 9.842, *t*-value: 4.249). Similar patterns are observed in academic promotion, interpersonal interactions, work environment, and compensation, with professors consistently outperforming lecturers in terms of satisfaction. The disparity reaches its apex in Total Satisfaction, where professors report a mean of 90.408 against lecturers’ 84.277, substantiated by a *t*-value of 15.471. The statistical significance of these differences is consistently marked by value of *p*s < 0.001, unequivocally indicating that university professors experience a higher degree of job satisfaction compared to lecturers, thereby substantiating the hypothesis.

**Table 5:** *T*-value analysis of job satisfaction by academic rank.

Dimensions	Academic rank	*N*	Mean	*SD*	*T*-value	*p*-value
Decision-making participation	Professor	216	16.408	1.619	3.967	<0.001
	Lecturer	105	15.614	0.836		
Teaching responsibilities	Professor	216	18.980	1.601	5.293	<0.001
	Lecturer	105	17.400	1.772		
Academic advising	Professor	216	11.601	2.145	4.249	<0.001
	Lecturer	105	9.842	1.347		
Academic promotion	Professor	216	6.327	6.889	3.248	<0.001
	Lecturer	105	6.050	0.357		
Interpersonal interactions	Professor	216	8.449	0.937	2.332	<0.001
	Lecturer	105	7.990	1.212		
Work environment	Professor	216	16.612	1.605	3.174	<0.001
	Lecturer	105	15.980	0.836		
Compensation	Professor	216	12.571	2.198	4.221	<0.001
	Lecturer	105	11.406	1.185		
Total Satisfaction	Professor	216	90.408	2.169	15.471	<0.001
	Lecturer	105	84.277	2.482		

The results presented in Table 6 affirm *Hypothesis 3*, showing a significant correlation between psychological resilience and job satisfaction among faculty members. Decision-making participation is highly correlated with total resilience (0.454), suggesting a strong association. Teaching responsibilities and academic advising both show significant correlations with total resilience (0.320 and 0.309, respectively), with academic advising also notably linked to the control dimension (0.437). Academic promotion correlates significantly with the challenge dimension (0.365), indicating the importance of resilience in this aspect. Interpersonal interactions display significant correlations across all resilience dimensions (commitment 0.225, control 0.282, challenge 0.458) and total resilience (0.205), highlighting resilience’s comprehensive impact. Work environment and compensation also correlate significantly with commitment and control, underscoring their influence on satisfaction. Crucially, total satisfaction shows a highly significant correlation with all resilience dimensions and total resilience (0.590), emphasizing the robust link between overall psychological resilience and job satisfaction.

**Table 6:** Correlation coefficients between psychological resilience and job satisfaction.

Dimensions	Commitment	Control	Challenge	Total resilience
Decision-making participation	0.060	0.021	0.126	0.454^**^
Teaching responsibilities	0.058	0.105	0.042	0.320^**^
Academic advising	0.007	0.437^**^	0.037	0.309^**^
Academic promotion	0.041	0.033	0.365^**^	0.037
Interpersonal interactions	0.225^**^	0.282^**^	0.458^**^	0.205^*^
Work environment	0.204^*^	0.205^*^	0.330	0.098
Compensation	0.355^**^	0.291^**^	0.013	0.009
Total Satisfaction	0.505^**^	0.332^**^	0.495^**^	0.590^**^

^**^Correlation is significant at the 0.01 level (2-tailed). ^*^Correlation is significant at the 0.05 level (2-tailed).

The analytical outcomes from Tables 7, 8 provide robust empirical support for *Hypothesis 4*, which hypothesized that the degree of job satisfaction among faculty members in Saudi Arabia could be effectively predicted by examining their psychological resilience. Table 7, utilizing a simple linear regression ANOVA test, reveals a significant model with an *F*-value of 7.683 and a value of p less than 0.001, indicating that psychological resilience, as an aggregate construct, significantly predicts job satisfaction levels. Advancing to Table 8, the stepwise multiple regression analysis elucidates this predictive relationship in greater detail. The specific dimensions of psychological resilience, namely commitment, control, and challenge, demonstrate significant predictive relationships with job satisfaction. Commitment, with a correlation coefficient (*R*) of 0.339 and explaining 15.9% of the variance in job satisfaction (*R*^2^ = 0.159), along with control (*R* = 0.315, *R*^2^ = 0.099) and challenge (*R* = 0.285, *R*^2^ = 0.081), exhibit noteworthy predictive capabilities, as evidenced by their significant *t*-values and *p*-values below 0.001. Collectively, these findings from both tables coherently substantiate the hypothesis, accentuating that the dimensions of psychological resilience are critical in predicting and understanding job satisfaction levels within the academic milieu of Saudi Arabia.

**Table 7:** Job satisfaction prediction from psychological resilience *p*-value.

Source of variance	Sum of squares	Degrees of freedom	Mean sum of squares	*F*-value	*p*-value
Regression	28.192	3	9.397	7.683	< 0.001
Error	178.581	146	1.223		
Total	206.773	149			

**Table 8:** Psychological resilience dimensions as predictors of job satisfaction.

Dimensions	*R*	*R* ^2^	Adjusted *R*^2^	*B*	*SE*	*β*	*T*-value	*p*-value
Commitment	0.339	0.159	0.150	0.154	0.042	0.294	3.675	< 0.001
Control	0.315	0.099	0.095	0.173	0.051	0.269	3.391	< 0.001
Challenge	0.285	0.081	0.079	0.096	0.046	0.163	3.089	< 0.001

β, standardized regression coefficients; B, unstandardized regression coefficients; SE, standard error of estimate; R, correlation coefficient.

The empirical evidence from Table 9 decisively validates *Hypothesis 5*, which postulated a significant correlation between psychological resilience and motivation in scientific research. The data reveals a notable correlation of accomplishment motivation with commitment (0.187), and even more pronounced correlations with control (0.394), challenge (0.316), and total resilience (0.336), particularly underscoring the significant role of the control dimension. In the ambit of ambition level, a consistent and highly significant correlation is observed across all resilience dimensions: commitment (0.352), control (0.244), challenge (0.299), and total resilience (0.284), indicating a comprehensive and robust linkage with overall psychological resilience. The challenge-seeking dimension further corroborates this pattern, exhibiting highly significant correlations with each aspect of resilience (commitment 0.259, control 0.411, challenge 0.336, and total resilience 0.412), with particular emphasis on control and total resilience. Furthermore, total motivation reflects strong and significant correlations with all resilience measures (commitment 0.243, control 0.427, challenge 0.343, and total resilience 0.419), reinforcing the pivotal influence of psychological resilience in catalyzing overall motivation for engaging in scientific research endeavors.

**Table 9:** Correlation coefficients between psychological resilience and scientific research motivation.

Dimensions	Commitment	Control	Challenge	Total resilience
Accomplishment motivation	0.187^*^	0.394^**^	0.316^**^	0.336^**^
Ambition level	0.352^**^	0.244^**^	0.299^**^	0.284^**^
Challenge seeking	0.259^**^	0.411^**^	0.336^**^	0.412^**^
Total motivation	0.243^**^	0.427^**^	0.343^**^	0.419^**^

^**^Correlation is significant at the 0.01 level (2-tailed). ^*^Correlation is significant at the 0.05 level (2-tailed).

In Table 10, the data robustly substantiates *Hypothesis 6*, which posited a significant nexus between job satisfaction and motivation for scientific research. Evidently, decision-making participation exhibits highly significant correlations with accomplishment motivation (0.775), ambition level (0.600), and total motivation (0.787), delineating a strong association with research motivation. Teaching responsibilities and academic advising mirror this trend, particularly evident in their correlation with total motivation (0.752 for both). Additionally, academic promotion maintains significant linkages, notably with challenge seeking (0.655). The dimensions of interpersonal interactions and the work environment are also significantly correlated with accomplishment motivation (0.657 and 0.729, respectively). Most pronounced is the correlation of total satisfaction with accomplishment motivation (0.830) and total motivation (0.832), highlighting a profound and consistent relationship between overall job satisfaction and the impetus for engaging in scientific research. This comprehensive set of findings affirms the substantial interconnection between various facets of job satisfaction and the dimensions of research motivation.

**Table 10:** Correlation coefficients between job satisfaction and scientific research motivation.

Dimensions	Accomplishment motivation	Ambition level	Challenge seeking	Total motivation
Decision-making participation	0.775^**^	0.600^**^	0.693^**^	0.787^**^
Teaching responsibilities	0.716^**^	0.624^**^	0.638^**^	0.752^**^
Academic advising	0.742^**^	0.587^**^	0.649^**^	0.752^**^
Academic promotion	0.698^**^	0.498^**^	0.655^**^	0.705^**^
Interpersonal interactions	0.657^**^	0.513^**^	0.577^**^	0.665^**^
Work environment	0.729^**^	0.540^**^	0.610^**^	0.715^**^
Compensation	0.725^**^	0.487^**^	0.557^**^	0.673^**^
Total satisfaction	0.830^**^	0.640^**^	0.717^**^	0.832^**^

^**^Correlation is significant at the 0.01 level (2-tailed).

## 5 Discussion and conclusion

This study investigates the complex interplay between job satisfaction, psychological resilience, and motivation in scientific research among faculty members in Saudi Arabia. It is essential for understanding their combined impact on academic performance and faculty well-being. The central research question delves into how these factors interact within the academic context. The researcher hypothesizes that the complexity of the academic environment is likely reflected in moderate levels of job satisfaction among faculty members. Furthermore, the study anticipates significant correlations: one between psychological resilience and job satisfaction, and another between resilience and motivation. This implies a dual role for resilience, not only in mitigating dissatisfaction but also in enhancing motivation for scientific inquiry. The investigation also extends to examining variations in job satisfaction across different academic ranks and exploring whether resilience can serve as a predictor of job satisfaction levels. Through these hypotheses, the study aims to unravel the intricate dynamics of job satisfaction, resilience, and motivation in academia, thereby providing insights into the experiences of faculty members within the Saudi Arabian academic landscape.

Moving on to the findings, the study confirmed its first hypothesis, positing moderate job satisfaction levels among faculty members in Saudi Arabian universities. This aligns with the nuanced perspectives on academic job satisfaction discussed in a study by Anasi (2020), which highlights the intricate interplay of emotional, cognitive, and behavioral factors in job satisfaction and the balance between job demands and personal fulfillment. Furthermore, the influence of internal and external motivational factors on job satisfaction, as indicated in the research by Szromek and Wolniak (2020), is evident in these findings. Mgaiwa (2021) suggests that job satisfaction stems from the perception of how well the job fulfills material and psychological needs, focusing on the alignment of work returns with personal desires and lifestyle.

Further exploring variations within the academic community, the research provides definitive data supporting *Hypothesis 2*, highlighting significant differences in job satisfaction levels between university professors and lecturers. This disparity, evident across various job aspects, suggests that professors may experience a more conducive work environment, potentially enhancing their job performance and engagement. This advantage for professors is reflected in their higher mean satisfaction scores compared to lecturers, aligning with Mgaiwa’s (2023) findings of greater career stability and satisfaction among professors. Shrestha (2019) further indicates a correlation between faculty members’ age and job satisfaction, suggesting that with increasing age and academic status, faculty members often develop a more pragmatic approach, leading to higher contentment in their roles. This trend is supported by Mystkowska-Wiertelak (2022), who observes that the alignment of professional aspirations with reality often improves over time. Additionally, as Mgaiwa (2023) notes, higher academic ranks usually come with greater knowledge and expertise, factors contributing to increased job satisfaction. These findings underscore the complex relationship between academic rank, age, and job satisfaction, highlighting the need for a nuanced understanding of faculty members’ experiences in higher education.

Building on these insights, this study confirmed *Hypothesis 3*, finding a significant correlation between psychological resilience and job satisfaction among academic faculty members. Job satisfaction aspects are strongly correlated with psychological resilience, as indicated by a composite score across multiple resilience dimensions. These results are consistent with prior research, such as Nagoji and Mackasare (2023), which explored the complex relationship between job satisfaction and resilience in academia. The researcher attributes these outcomes to the role of psychological resilience in enhancing faculty members’ engagement and effectiveness at work, as observed by Brewer et al. (2019); their proficiency in managing professional stress, as noted by Yang et al. (2022); and their increased job satisfaction and adaptability in handling challenges, per Kowler et al. (2023).

Continuing this trajectory, the current study affirms *Hypothesis 4*, revealing a significant link between psychological resilience and job satisfaction among faculty members in Saudi Arabia. This finding is in line with Singh et al.’s (2022) research, which identified a correlation between psychological stress, burnout, job dissatisfaction, and reluctance to work in academia. García-Rivera et al. (2022) further support this by showing the critical role of the challenge dimension, a key aspect of psychological resilience, in explaining variations in faculty job satisfaction. These results resonate with Gundogan’s (2021) perspective that psychological resilience is a key determinant of overall life satisfaction, which Genç (2023) expands by noting its connection to an individual’s psychological well-being, social, and functional adjustment. This underscores the comprehensive impact of psychological resilience on various life aspects, particularly within the academic profession.

This study provides robust support for the proposed significant relationship between psychological resilience and motivation in academic contexts, as evidenced by the empirical findings. A noteworthy correlation was observed between different dimensions of resilience and motivation; specifically, the control dimension of resilience showed a positive correlation with achievement motivation and challenge-seeking. Moreover, the overall resilience score was positively correlated with general motivation levels, underscoring the quantitative impact of resilience on motivational factors crucial to academic productivity and engagement. The positive psychology literature, especially the work of Boyce-Tillman (2021), which stress psychological resilience as a dynamic force improving life quality and a major contributor to faculty members’ achievements, adds to the importance of these findings. The recent study by Sawitri et al. (2023) expands this understanding by positioning resilience not only as a coping mechanism but also as a central catalyst for motivation, thus enriching the current research landscape on the multifaceted role of resilience in the academic environment.

Finally, the study’s findings support *Hypothesis 6*, revealing a significant link between job satisfaction and motivation in scientific research. Specifically, higher levels of job satisfaction in areas like decision-making and teaching duties were associated with increased research motivation. This correlation, particularly strong in decision-making satisfaction, aligns with previous research by Atieno et al. (2022) and Mudrák et al. (2020), which highlighted job satisfaction as a key driver of professional motivation. Ismayilova and Klassen (2019) also underscore the role of job satisfaction in enhancing research motivation and organizational commitment among faculty members. The researcher attributes this outcome to the fact that job satisfaction, characterized by engaging in enjoyable work and receiving recognition, directly influences a faculty member’s sense of achievement and success (Nelson et al., 2020). This interpretation aligns with the return on equity theory, as proposed by Beuren et al. (2022), suggesting that job satisfaction hinges on the perceived return and advancement from one’s work. Thus, job satisfaction in higher education is a crucial indicator of academic success, encapsulating the positive emotions and attitudes of faculty members towards their roles in academic institutions.

### 5.1 Theoretical implications

This study offers significant theoretical implications. Firstly, the research resonates with and extends the AET, emphasizing the role of emotional aspects in shaping job perceptions in academia. The correlation between psychological resilience and job satisfaction enhances AET’s relevance (Ghasemy et al., 2022b), particularly in the unique cultural context of Saudi Arabia. Concurrently, these findings align with SDT, underscoring psychological resilience as a crucial intrinsic factor in academic motivation. This extension of both AET and SDT highlights the importance of personal resilience in the academic landscape (Hanson et al., 2022).

Furthermore, this study offers a comprehensive analysis of Herzberg’s two-factor theory of job satisfaction, with a specific focus on its application within the academic sector. The variations in levels of job satisfaction among different academic ranks indicate a multifaceted interaction between internal and extrinsic elements, which poses a challenge to the conventional simplicity of Herzberg’s model and Maslow’s hierarchical approach. This intricacy underscores the need for a comprehensive comprehension of faculty contentment, considering a wide range of requirements and anticipations inside academic positions, particularly within the framework of Saudi Arabian higher education institutions.

Additionally, this study integrates and expands upon the SCCT and the JCM. The research emphasizes the significance of maintaining a harmonious relationship between personal resilience, an inherent characteristic, and the external academic environment, which is consistent with the SCCT. Also, the fact that different academic ranks are affected by work characteristics like autonomy and professional growth in different ways shows how complicated it is to use the JCM in an academic setting. In line with the integrative framework suggested by Stupnisky et al. (2023), this study also suggests that hierarchical needs, job characteristics, and intrinsic factors like psychological resilience all play a role in how satisfied and motivated faculty members are in the academic setting. This highlights the importance of adopting a comprehensive perspective, particularly in the context of Saudi Arabian universities.

### 5.2 Practical implications

In light of the study’s findings on job satisfaction and psychological resilience among faculty members in Saudi Arabian universities, it becomes imperative to translate these insights into actionable strategies. At the institutional level, a primary focus should be on developing policies and programs that bolster faculty resilience and mental well-being. This encompasses implementing robust support systems for mental health, encompassing counseling services, stress management workshops, and peer support initiatives. Simultaneously, enhancing faculty development through targeted training programs that emphasize emotional intelligence and resilience skills is crucial. Such initiatives not only aid in equipping faculty members to handle the dynamic and often challenging academic environment but also foster a culture of continuous learning and adaptation. This dual approach of personal development and institutional support forms the cornerstone of fostering a resilient, satisfied, and motivated academic workforce, tailored to the unique cultural and operational milieu of Saudi Arabian higher education institutions.

Equally important is the need to acknowledge and adapt to the unique cultural context of Saudi Arabian universities, which plays a significant role in shaping faculty job satisfaction and motivation. This involves not only recognizing cultural nuances but also actively incorporating them into institutional policies and faculty engagement strategies. Enhancing faculty autonomy in decision-making processes, particularly in areas that directly affect their work and research, can lead to increased job satisfaction and a sense of ownership. Additionally, the establishment of effective recognition and reward systems that go beyond traditional financial incentives is vital. These systems should celebrate academic achievements, resilience in facing challenges, and contributions to the university community. By integrating cultural understanding with increased autonomy and a robust recognition system, universities can create an environment where faculty members feel valued, understood, and motivated to excel in their roles.

Furthermore, the well-being of faculty members extends beyond professional development and recognition, necessitating a holistic approach that includes comprehensive wellness programs and a supportive research environment. Establishing wellness initiatives that cater to both physical and psychological health can significantly impact faculty morale and productivity. These initiatives might include regular health screenings, fitness classes, and mindfulness sessions, which collectively contribute to a healthier, more balanced academic community. In parallel, ensuring adequate resources and support for research is critical. This means not only improving the research infrastructure but also fostering a culture that values and facilitates scholarly inquiry. Additionally, building a supportive and collaborative community among faculty members can enhance job satisfaction. Initiatives such as mentorship programs, interdisciplinary collaborations, and social events can strengthen community bonds and provide a nurturing environment for both personal and professional growth. This comprehensive approach, which integrates wellness programs, research support, and community building, is essential for creating an academic environment where faculty can thrive and feel a profound sense of belonging and achievement.

### 5.3 Limitations and future studies

This study provides valuable insights into job satisfaction and psychological resilience among faculty members in Saudi Arabian universities but has certain limitations that pave the way for future research opportunities. A primary limitation is its focus on Saudi Arabian universities, which may limit the generalizability of its findings. This suggests the need for future comparative studies in varied cultural and geographical contexts. Moreover, the reliance on mainly quantitative methods might not fully capture the depth of faculty members’ subjective experiences, highlighting the potential benefit of qualitative or mixed-methods research. The exclusion of specific sociodemographic factors like tenure and marital status limits the depth of demographic analysis, indicating a need for more comprehensive future studies.

Additionally, while the current study effectively captures the correlations among key variables using SPSS, employing advanced statistical techniques like mediation analysis would allow a more nuanced exploration of these relationships. However, these methods were beyond this study’s scope. Future research integrating such approaches could reveal mediator variables that further explain the dynamics between psychological resilience, job satisfaction, and research motivation. For instance, work-life balance could be examined as a mediator, and academic rank as a moderator. Furthermore, the use of newly developed scales, while methodologically sound, could be further validated using advanced statistical programs like AMOS or SmartPLS. Lastly, given the evolving nature of job satisfaction and psychological resilience, longitudinal research could offer insights into how these aspects change over time, especially in response to institutional interventions or academic landscape shifts. These avenues present an opportunity to build upon this study’s foundational work, enhancing understanding and application in a global context.

## Data availability statement

The raw data supporting the conclusions of this article will be made available by the authors, without undue reservation.

## Ethics statement

The studies involving humans were approved by University of Business and Technology Research Ethical Committee. The studies were conducted in accordance with the local legislation and institutional requirements. The participants provided their written informed consent to participate in this study. Written informed consent was obtained from the individual(s) for the publication of any potentially identifiable images or data included in this article.

## Author contributions

AA: Conceptualization, Data curation, Formal analysis, Funding acquisition, Investigation, Methodology, Project administration, Resources, Software, Supervision, Validation, Visualization, Writing – original draft, Writing – review & editing.
